# Opportunities and challenges for antisense oligonucleotide therapies

**DOI:** 10.1002/jimd.12251

**Published:** 2020-06-03

**Authors:** Elsa C. Kuijper, Atze J. Bergsma, W.W.M. Pim Pijnappel, Annemieke Aartsma‐Rus

**Affiliations:** ^1^ Department of Human Genetics Leiden University Medical Center Leiden The Netherlands; ^2^ Department of Pediatrics Center for Lysosomal and Metabolic Diseases, Erasmus Medical Center Rotterdam The Netherlands; ^3^ Department of Clinical Genetics Center for Lysosomal and Metabolic Diseases, Erasmus Medical Center Rotterdam The Netherlands

**Keywords:** antisense oligonucleotides, personalized medicine, RNA therapeutics, splicing modulation, targeted gene knockdown, therapies

## Abstract

Antisense oligonucleotide (AON) therapies involve short strands of modified nucleotides that target RNA in a sequence‐specific manner, inducing targeted protein knockdown or restoration. Currently, 10 AON therapies have been approved in the United States and Europe. Nucleotides are chemically modified to protect AONs from degradation, enhance bioavailability and increase RNA affinity. Whereas single stranded AONs can efficiently be delivered systemically, delivery of double stranded AONs requires capsulation in lipid nanoparticles or binding to a conjugate as the uptake enhancing backbone is hidden in this conformation. With improved chemistry, delivery vehicles and conjugates, doses can be lowered, thereby reducing the risk and occurrence of side effects. AONs can be used to knockdown or restore levels of protein. Knockdown can be achieved by single stranded or double stranded AONs binding the RNA transcript and activating RNaseH‐mediated and RISC‐mediated degradation respectively. Transcript binding by AONs can also prevent translation, hence reducing protein levels. For protein restoration, single stranded AONs are used to modulate pre‐mRNA splicing and either include or skip an exon to restore protein production. Intervening at a genetic level, AONs provide therapeutic options for inherited metabolic diseases as well. This review provides an overview of the different AON approaches, with a focus on AONs developed for inborn errors of metabolism.

## 1 INTRODUCTION

Antisense oligonucleotide (AON) therapies are coming of age; currently 10 AONs have been approved by the Food and Drug Administration (FDA, USA), and/or the European Medicines Agency (EMA) and/or the Japanese Ministry of Health, Labour and Welfare, the majority of which obtained marketing authorization in the past 4 years (Table 1). This therapeutic approach uses small pieces of modified DNA or RNA that are synthesized from chemically modified nucleotides.^11^, ^12^ They target RNA in a sequence specific manner through Watson‐Crick base pairing, and can induce targeted protein knockdown or protein restoration. Compared to chemical compounds, antisense oligonucleotide therapies have unprecedented specificity, for example, they provide the possibility to target specific transcript isoforms or a specific member in a family of closely related proteins. Because they intervene at a genetic level, they provide therapeutic options particularly for inherited diseases. In this review we will give a high level overview on therapeutic AONs, including modifications needed to endow them with drug‐like properties, delivery and safety considerations, and provide examples of currently approved antisense oligonucleotides. Finally, we will outline how these modalities have been explored to treat inherited metabolic diseases.

**TABLE 1:** Overview of approved antisense oligonucleotide drugs

Compound	Disease (OMIM number)	Mechanism of action	Delivery route	Approved by (when)	Reference
Fomivirsen	CMV‐induced retinitis (NA)	Formivirsen binds to UL123 transcripts and prevents translation into IE2 protein	Intraocular	FDA (1998), EMA (1999)	^1^
Mipomirsen	Familial hypercholesterolemia (607748)	Mipomirsen binds to ApoB‐100 transcripts and induces RNase H mediated cleavage of targeted transcripts	Subcutaneous	FDA (2013)	^2^
Eteplirsen	Duchenne muscular dystrophy (310200)	Eteplirsen binds to exon 51 in pre‐mRNA DMD transcripts, thus preventing inclusion in mature mRNA; this restores the reading frame allowing the production of partially functional dystrophin protein	Intravenous	FDA (2016)	^3^
Nusinersen	Spinal muscular atrophy types I, II and III (25330, 253550, 253400)	Nusinersen binds to an intronic splicing silencer in SMN2 pre‐mRNA, thus enhancing SMN2 exon 7 inclusion and increasing production of SMN protein	Intrathecal	FDA (2016) EMA (2017)	^4^
Inotersen	Hereditary transthyretin‐mediated amyloidosis (105210)	Inotersen binds to hATTR transcripts and induces RNase H mediated cleavage, thus reducing thransthyretin protein production	Subcutaneous	FDA (2018) EMA (2018)	^5^
Patisiran	Hereditary transthyretin‐mediated amyloidosis (105210)	Patisiran is incoorporated into the RISC complex and induces si‐RNA mediated reduction of hATTR transcripts and thransthyretin protein production	Intravenous	FDA (2018) EMA (2018)	^6^, ^7^
Volanesorsen	Hypertriglycidemia (145750), familial chylomicronemia syndrome (118830) and familial partial lipodystrophy (151660)	Volanesorsen binds to apolipoprotein C‐III transcripts and induces RNase H induced cleavage and reduced protein formation; used in combination with low fat diet	Subcutaneous	EMA (2019)	^8^
Givosiran	Acute hepatic porphyrias (612740 and 176000)	Givosiran is incorporated into the RISC complex and induces si‐RNA mediated reduction of ALAS1 transcripts and reduced formation of toxic heme intermediates	Subcutaneous	FDA (2019)	^9^
Golodirsen	Duchenne muscular dystrophy	Golodirsen binds to exon 53 in pre‐mRNA DMD transcripts, thus preventing inclusion in mature mRNA; this restores the reading frame allowing the production of partially functional dystrophin protein	Intravenous	FDA (2019)	
Viltolarsen	Duchenne muscular dystrophy	Viltolarsen binds to exon 53 in pre‐mRNA DMD transcripts, thus preventing inclusion in the mature mRNA; this restores the reading frame allowing the production of partially functional dystrophin	Intravenous	Japanese Ministry for Health, Labour and Welfare (2020)	^10^

## 2 ANTISENSE OLIGONUCLEOTIDE DRUG CONSIDERATIONS

### 2.1 Chemical modifications

Therapeutic AONs are generally 15‐30 nucleotides long and can be single or double‐stranded moieties. Short DNA and RNA molecules are very sensitive to degradation by endo‐ and exonucleases. As such, AONs require chemical modifications to increase nuclease resistance.^11^, ^13^ These modifications can also provide additional benefits to the AONs, such as increased affinity to the target transcripts or improved bioavailability. Many different chemical modifications are currently available. The first and most widely used modification is the phosphorothioate (PS) modification, where an oxygen in the AON backbone is replaced by a sulfur atom.^14^ Although this modification reduces the affinity of the AON to its target, it improves stability, uptake and bioavailability. AONs are compounds of intermediate molecular weight (5‐12 kDa), meaning they are small enough to be filtered out by the kidney. AONs with a PS backbone will, however, bind serum proteins with low affinity, thus preventing renal clearance and improving uptake by other organs after systemic delivery, in particular the liver.

Additional modifications to the sugar ribose (2′*O*‐methyl and 2′*O*‐methoxyethyl, 2OMe and MOE, respectively) increase affinity to the target RNA and reduce some of the PS induced toxicity (see below). Finally, some modifications change the whole nucleotide, such as the phosphorodiamidate morpholino oligomers (PMO) or peptide nucleic acids (PNA). These modifications show little resemblance to the original nucleotides and as such are not recognized by, for example, nucleases. However, they maintain the ability to target transcripts in a Watson‐Crick manner. Which modifications to use varies depending on the AON modality used and the target tissue,^11^, ^12^, ^13^, ^15^ as we describe later.

### 2.2 Delivery

For single stranded AONs with a PS backbone systemic delivery is feasible. The majority of AON will be taken up by liver and kidney, but some uptake by most other tissues is occurring, with the exclusion of the nervous system, as most AON chemistries are unable to cross the blood‐brain barrier. An exception is the tricyclo‐PS modification, where a minute amount of compound appears to reach the central nervous system (CNS) when large amounts are injected systemically.^16^ Notably, after local delivery of single stranded MOE‐PS AONs to the CNS, intracerebroventricular in mice and intrathecal in non‐human primates and humans, these AONs are rapidly taken up by the neurons and distribute throughout the CNS.^17^, ^18^ For systemic delivery of single stranded AONs often a frequent dosing regimen is used with multiple injections per month. By contrast, the half‐life of AONs in the CNS is much longer; in the order of months in non‐human primates and humans, which enables less frequent treatment.

For double stranded AONs delivery is more challenging. The PS backbone is sheltered in the double stranded composition, thus limiting its potency as an uptake enhancer. However, it has been possible to have efficient uptake by hepatocytes through capsulation of double stranded AONs in lipid nanoparticles.^19^ Even more efficient liver uptake can be achieved with the GalNac conjugate.^20^ This binds specifically to the asialoglycoprotein receptor that is highly abundant on liver cells, and able to generate receptor‐mediated uptake of its ligand quickly and efficiently, with a recycle time of ~15 minutes.

### 2.3 Safety

There are two types of safety considerations for AON therapies. First, there are sequence specific safety aspects. For AON strategies aiming at protein knockdown, exaggerated pharmacology can be an issue, where too much knockdown of the target transcripts and encoded proteins in the target tissue, or the nontarget tissue can lead to undesired effects. In addition, it is possible that the AON binds to nontarget transcripts. The latter can often be avoided using in silico screening to confirm uniqueness of the AON target in the human genome or transcriptome. However, some AON chemistries have a very high affinity for RNA, and may also bind promiscuously to other transcripts, despite one or more mismatches. Searching for a target sequence that is as unique as possible can reduce this risk.

The second safety aspect is related to the chemical modifications of the AONs.^11^, ^12^ Different modifications have their own safety profile, where AONs of the same chemistry often behave as a class, with the exceptions of some motifs that are known to induce an immune response through activation of Toll‐like receptors.^21^ Often CpG motifs are involved in this, and methylating the “C” nucleotides of the AONs generally negates this effect to a great extent.^22^, ^23^ Furthermore, one can screen for the occurrence of these effects in vitro, thus deselecting AONs that induce an immune response.

Overall, the PS backbone is the main driver of toxicity. For a more elaborate outline of these effects we refer the reader to review papers on this topic.^24^, ^25^ In summary, through specific binding of serum proteins AONs with a PS backbone can inhibit coagulation (by inhibition of the tenase pathway) and activate complement (through Factor H binding).^25^, ^26^, ^27^ In addition, there are safety findings related to sites where AONs accumulate, that is, liver, kidney, and lymph nodes.^24^ Proteinuria is frequently observed in patients who are treated systemically and chronically with AONs, probably due to accumulation of AONs in the proximal tubuli, sometimes inducing apoptosis and hence interfering with protein reabsorption. In the liver AONs are mainly taken up by the Kupffer cells and hepatocytes. In lymph nodes AON accumulation can result in an inflammatory response. Subcutaneous delivery of PS‐modified AONs result in injection site reactions due to an as yet unknown mechanism. Finally, in a subset of patients thrombocytopenia has been reported to occur,^24^, ^28^ resulting in hospitalization of patients and possibly death for one patient.^5^ This risk of thrombocytopenia appears to be sequence and dose dependent.^29^ With improved chemistry, delivery vehicles and conjugates, patients can now be dosed a lower levels, thus reducing the risk and occurrence of side effects.

## 3 HOW TO EXPLOIT ANTISENSE OLIGONUCLEOTIDE DRUGS FOR GENETIC DISEASES

**FIGURE 1:**
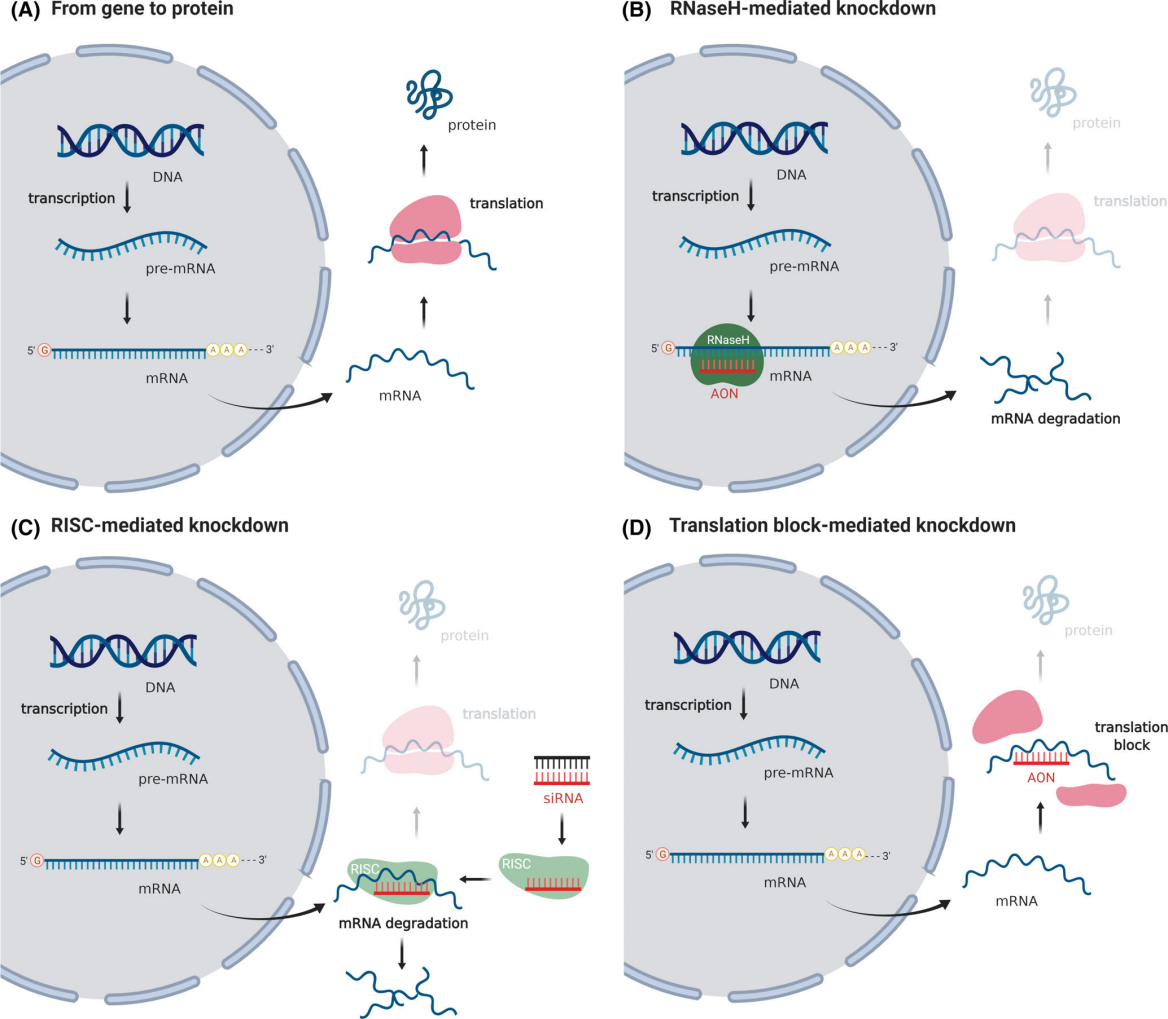
AON‐mediated protein knockdown. A, The pre‐mRNA transcribed from the DNA is spliced and capped to obtain the final mRNA, which is exported to the cytoplasm and translated into protein at the ribosomes. B, As the single stranded AON binds to the mRNA, this complex is recognized and degraded by RNaseH, an endonuclease present in nucleus and cytoplasm, blocking protein production. C, From the double stranded siRNA, the guide strand is incorporated into the RNA‐induced silencing complex (RISC). This complex specifically binds the targeted mRNA, inducing its degradation and inhibiting protein production. D, The AON binds the mRNA, thereby changing its conformation, preventing the formation of the ribosome and blocking the process of protein translation

**FIGURE 2:**
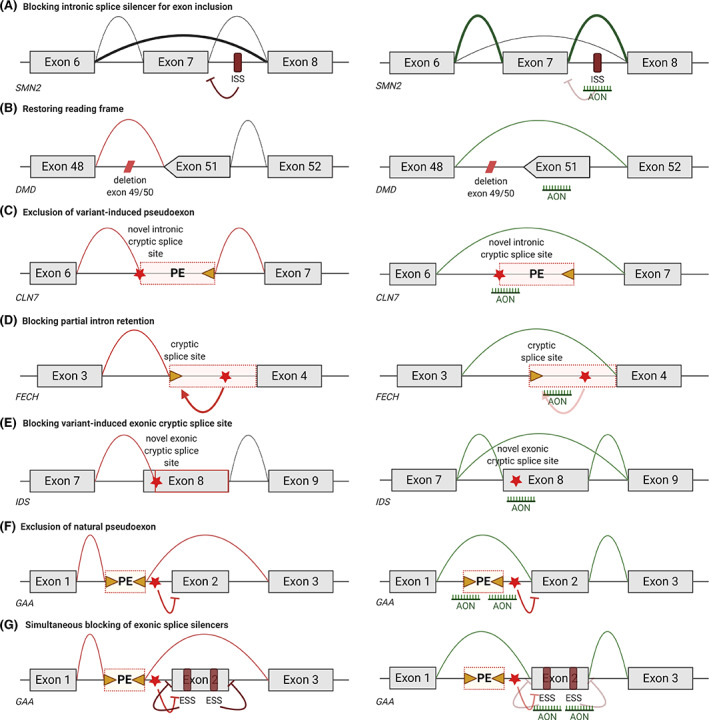
Schematic depiction of AON‐mediated protein restoration strategies using on disease examples. A, Exon inclusion. Normally, the majority of SMN2 transcripts does not include exon 7 which prevents the production of functional protein. By blocking an intronic splice silencer that prevents recognition of exon 7, AON treatment stimulates the inclusion of exon 7 and thereby production of functional protein. B, Exon skipping. The deletion of exons 49 and 50 disrupts the DMD open reading frame leading to a premature stop codon. AON‐mediated skipping of exon 51 restores the reading frame and allows production of partially functional protein. C, Restoring pseudoexon inclusion. When a variant creates a novel intronic cryptic splice site, a pseudoexon can be included in the CLN7 transcript. Blocking of this cryptic splice site by AONs restores normal splicing. D, Restoring partial intron retention. A variant‐induced intronic cryptic splice site leads to partial intron retention of FECH as this splice site is now used as splice acceptor site. Blocking of the cryptic splice acceptor site restores normal splicing. E, Restoring cryptic splicing. In case of a variant‐induced exonic cryptic splice site as in IDS, part of the exon is excluded as the cryptic splice site is used. AON treatment prevents the used of the cryptic splice site and stimulates use of the canonical splice site. F, Modulating alternative splicing. A variant in GAA silences the normal splice acceptor site of exon 2, thereby enabling the inclusion of a natural pseudoexon. Blocking of the pseudoexon splice sites with AONs restores normal splicing. G, Another way to prevent the inclusion of a pseudoexon in GAA is by strengthening the canonical splice sites of exon 2 by blocking of exonic splice silencers in exon 2 with AONs. Note this figure shows several but limited examples of protein restoring strategies. Star, variant; triangle, cryptic splice site

AONs can be used to modulate gene expression by targeting transcripts. First, AONs can be used to achieve targeted knockdown of a toxic protein or a key protein in a pathological pathway (Figure 1). It is also possible to restore production of a missing protein by AON‐induced splicing modulation (Figure 2). Notably, as a genetic therapy approach AONs differ in several ways from gene addition approaches. First, AONs target gene transcripts, while with gene therapy, generally cDNA of a missing or mutated gene is provided. Due to turnover of AONs, effects are transient, while for gene addition therapies generally effects have a more permanent nature. AONs are short, synthetic pieces of modified RNA or DNA, which are mostly delivered without an excipient. Gene addition therapies mostly rely on viral vectors to deliver transgenes that can be thousands of bp long.

Here we will outline the different AON modalities focusing on those that have been marketed so far.

### 3.1 Protein knockdown

Single stranded AONs can induce targeted knockdown of transcripts by activating RNase H, an enzyme that cleaves DNA:RNA hybrids^30^(Figure 1B). Since RNase H‐mediated cleavage is initiated at the 2′ position of the ribose, modifications at this position (eg, 2OMe, MOE) or modifications that significantly alter the nucleotide structure (eg, PMO, PNA) render AONs RNase H resistant. To still benefit from the increased affinity of the modified nucleotides, generally a gapmer AON is used, with a PS stretch in the middle that initiates RNase H cleavage, flanked by more modified nucleotides at both ends (Figure 1B). Examples of RNase H AONs are mipomersen and volanesorsen (Table 1).^8^, ^31^ These bind to Apo‐B 100 and apolipoprotein C‐III transcripts, respectively, which are both highly expressed in liver, one of the main target organs after systemic AON delivery. Subcutaneous treatment with mipomersen and volanesorsen has resulted in a significant reduction in cholesterol or triglyceride levels in blood of patients with familial high cholesterolemia or triglyceridemia, respectively. Mipomersen was approved in 2013 by the FDA, but was discontinued in 2018 due to limited clinical effects and safety concerns related to its hepatotoxicity.^32^

Double stranded siRNAs are another tool to achieve transcript knockdown^33^ (Figure 1C). siRNAs “hijack” the RNA‐induced silencing complex (RISC), where the guide strand of the siRNA is incorporated into the RISC and binds specifically to the target, which is then degraded. RISC induced silencing is a catalytic process and some siRNAs have been shown to result in functional effects that persists for months after treatment.^34^ The guide strand generally cannot be chemically modified, because this interferes with incorporation into the RISC complex. However, the opposite strand (passenger strand) does tolerate chemical modifications, to ensure stability of the siRNA.

Currently, two siRNA AON has been approved, patisiran and givosiran.^7^ Patisiran is an siRNA that targets transthyretin transcripts as a therapy for hereditary transthyretin (hATTR)‐mediated amyloidosis. These patients have variants in the *TTR* gene that lead to aggregation of transthyretin protein, resulting in a multisystemic disorder that includes polyneuropathy and cardiac problems. The primary expression site for transthyretin is liver hepatocytes. As mentioned, delivery of double stranded siRNAs to any tissue is challenging. Therefore, a lipid nanoparticle formulation is used to ensure sufficient patisiran delivery to and uptake by hepatocytes. In a clinical trial intravenous treatment with patisiran resulted in a significant and clinically relevant reduction of polyneuropathy.^6^ Notably, inotersen is a single stranded RNase H cleavage inducing AON that targets the same transcript as patisiran. Subcutaneous delivery of inotersen has also been approved by FDA and EMA for treatment of hATTR‐mediated amyloidosis based on a slower disease progression and improved quality of life for treated patients compared to placebo.^5^

Acute hepatic porphyria (OMIM#612740) is an inherited metabolic disorder caused by variants in the *ALAD* gene, that encodes the protein porphobilinogen synthase, a protein involved in hepatic heme synthesis. Defects in this gene results in accumulation of heme intermediates. Givosiran is an siRNA that targets delta aminolevulinic acid synthase 1 (ALAS1) transcripts, which reduces the production of toxic intermediates. In a clinical trial, subcutaneous givisoran treatment resulted in a significant reduction of porphyria attacks in treated patients, thus far without any sign of heme deficiency.^9^ Givisoran is the first approved oligonucleotide carrying a GalNac conjugate.

### 3.2 Protein restoration

Single stranded AONs that are fully RNase H resistant can be used to modulate pre‐mRNA splicing, the process where introns are removed from gene transcripts, and exons are joined to form the mRNAs^35^ (Figure 2).

The most notable example of a splice modulating is the exon inclusion achieved by nusinersen (Figure 2A), an intrathecally delivered MOEPS modified AON that is approved for all types of spinal muscular atrophy (SMA), a set of diseases characterized by the progressive loss of motorneurons and motorfunction, ranging from very severe (type I) to severe (type III) to the milder SMA type IV.^36^ SMA type I patients never are able to sit independently and generally die before the age of 2 years. SMA types II and III have a later onset and patients are originally able to sit (type II) or stand (type III), but also here patients suffer from progressive loss of motorneurons and motor function. Type IV is a rare, adult‐onset form of the disease with a milder progression. SMA is caused by variants in the *SMN1* gene that abolish production of survival motorneuron (SMN) protein.^37^ Complete lack of SMN is embryonically lethal. Primates carry a homologous *SMN2* gene, which allows the production of low levels of SMN protein. While the *SMN1* and *SMN2* genes are almost identical, small variations cause *SMN2* exon 7 to be very poorly recognized by the splicing machinery. Thus, only 10% of the transcripts include exon 7 and allow production of stable SMN protein. There is copy number variation for *SMN2*, and the number of *SMN2* gene inversely correlates with the severity of SMA.^36^

Nusinersen is an AON that binds to intron 7 of *SMN2* transcripts and thereby facilitates recognition and mRNA inclusion of exon 7 by the splicing machinery. Because SMA primarily affects the motorneurons, delivery to the nervous system is required. Intrathecal delivery of nusinersen has been tested in double‐blind, randomized placebo‐controlled clinical trials with SMA types 1 and II patients and in each case the primary endpoint was met already during interim analysis.^38^, ^39^ For SMA type I this involved event free survival, which was significantly increased compared to sham treated patients, while for SMA type II, the motor score was significantly improved compared to baseline, while for sham treated patients it declined. Since all types of SMA are characterized by progressive and irreversible loss of motorneurons, earlier treatment would be preferred. A currently ongoing trial where SMA patients are treated presymptomatically indeed suggests that earlier treatment results in more significant therapeutic effects.^40^ Recently, also an adeno‐associated viral vector‐mediated gene therapy (onasemnogene abeparvovec, tradename Zolgensma) has been approved for treatment of SMA patients by FDA and EMA. Now that multiple therapies are available for this disease and in light of the preference for early treatment, efforts to implement neonatal screening for SMA are ongoing in various countries.^41^

Splice modulating AONs can also be used to induce exon skipping, that is, remove an exon from the mRNA transcript^42^ (Figure 2B). Duchenne muscular dystrophy (DMD) is an early onset, severely progressive muscle wasting disease, caused by variants in the *DMD* gene that cause premature truncation of dystrophin protein translation. Dystrophin provides muscle fibers with stability by connecting the actin cytoskeleton to the extracellular matrix. Individuals with variants that do not disrupt the open reading frame, allowing the production of internally deleted dystrophins that maintain their connecting domains are affected by the less severe Becker muscular dystrophy. AON‐mediated exon skipping can be used to restore the reading frame of the DMD transcripts, allowing them to produce Becker‐type, partially functional dystrophins. This approach is variant specific. However, most DMD patients carry a large deletion of one or more exons, which cluster in a hotspot. As such, skipping certain exons applies to larger groups of patients. AONs inducing exon 51 skipping, which applies to 14% of patients, have been developed furthest.^42^ Because most of the over 700 skeletal muscles in the human body are affected by DMD, systemic treatment is required. Drisapersen, a 2OMePS AON, was evaluated in 300 DMD patients, and initially showed promising results in young DMD patients, but a phase 3 trials failed to meet its primary endpoint (6 minute walking distance) and development was abandoned.^3^ Eteplirsen, a PMO AON has received accelerated approval by the FDA, based on minute increases in dystrophin expression. Functional efficacy has not yet been confirmed and evidence for this needs to be provided to the FDA by 2021.^3^ Similarly, golodirsen and viltolarsen, both PMOs for exon 53 skipping received accelerated approval from FDA and conditional approval from the Japanese Ministry of Health, Labour and Welfare recently, based only on increases in dystrophin expression, while functional efficacy will need to be confirmed in the future.

AON‐mediated splicing modulation is also a very appealing approach for cryptic splicing variants (Figure 2C). These involve intronic variants that result in the inclusion of an intronic part of the transcript in the mRNA transcript (pseudo‐exon). When these pseudoexons are blocked by AONs, this may restore normal splicing and protein production (Figure 2C). The challenge, however, is that generally cryptic splicing variants are private, that is, occurring in only a single patient or family. Nevertheless, proof‐of‐concept of the feasibility of developing an AON treatment for an individual cryptic splicing variant was recently reported. This involved a patient with neuronal ceroid lipofuscinosis 7 (a type of Batten's disease, a severe progressive neurodegenerative disorder, OMIM#610951).^43^ In this case, the disease was caused by compound heterozygous *CLN7* variants, including one allele carrying a variant that resulted in inclusion of a pseudoexon. A team of researchers and clinicians managed to design an AON, test it in patient‐derived fibroblasts, have interaction with and advice from FDA and perform the required toxicity tests in rat, resulting in the initiation of an N = 1 trial in the patient within a year of diagnosis. Currently the patient receives four intrathecal injections of 42 mg “milasen,” a MOEPS AON, per year. So far the treatment is tolerated well. Since the initiation of the treatment functional scores, which were mainly declining prior to treatment, have mostly stabilized. Furthermore, a year after treatment onset, the patients suffers from between 0 and 20 seizures per day, which last less than a minute, where before treatment, she experienced 15‐30 seizures daily, which lasted 1‐2 minutes.

Additional efforts to develop AON therapies for individual or small groups of patients have been initiated at various locations. It is obviously impossible to conduct clinical trials for these AONs. Nevertheless, it will be important to measure efficacy in these patients in some way, for example, by assessing functional decline or evaluating whether the missing protein is restored if this can be measured in an easily accessible fluid or tissue.

## 4 ANTISENSE OLIGONUCLEOTIDES AS A POTENTIAL TREATMENT FOR INHERITED METABOLIC DISEASES

Approved therapies for the metabolic disorders familial hypercholesterolemia and acute hepatic porphyria have been discussed above. Clinical trials and preclinical studies are currently ongoing for the development of AON therapies for several additional metabolic disorders (Table 2) (previously reviewed by Perez et al^89^). These are discussed in more detail later.

**TABLE 2:** Overview of AON therapy development for inherited metabolic diseases

Disease (OMIM)	Target gene(s)	Therapeutic mechanism	Target	Backbone of AON	Stage of development	Trial numbers	EMA/FDA approval	Main affected tissue	Route of administration	Year	References
6‐Pyruvoyl‐tetrahydropterin synthase deficiency (261600)	PTPS	Exclusion of a variant‐induced pseudoexon	PTPS intron 1 and 2	PMO	Preclinical	N.a.	No	Brain	N.a.	2011	^44^
Acute hepatic porphyria (612740)	ALAS1	Targeted knockdown	ALAS1 exon 4	siRNA, mixed 2′‐fluoro and 2′‐O‐methyl‐PS	Approved drug, Givlaari (Givosiran)	NCT02452372, NCT03505853, NCT02949830, NCT04056481, NCT03338816	EMA, 2020 and FDA, 2019	Liver	Subcutaneous injection	2015, 2016, 2017, 2018	9, 45, 46
Canavan disease (271900)	Nat8l	Targeted knockdown	Nat8l exon 1	Gapmer (LNA)	Preclinical	N.a.	No	Brain	N.a.	2020	47
Congenital disorder of glycosylation type 1a (CDG‐1a) (212065)	PPM2	Exclusion of a variant‐induced pseudoexon	PPM2 intron 7	PMO	Preclinical	N.a.	No	Affects various tissues	N.a.	2009	48
Erythropoietic protoporphyria (177000)	FECH	Blocking of variant‐induced cryptic splice site	FECH intron 3	LNA	Preclinical	N.a.	No	Skin, liver	N.a.	2014	49
Fabry disease (301500)	GLA	Exclusion of a variant‐induced pseudoexon	GLA intron 4	2′‐O‐methyl‐PS	Preclinical	N.a.	No	Affects various tissues	N.a.	2016	50
Familial hypercholesterolemia (144010 and 607748)	APOB	Targeted knockdown	APOB exon 21 splice acceptor	Gapmer (2′‐O‐methoxyethyl, DNA)	Approved drug, Kynmamro (Mipomersen sodium)	NCT00216463, NCT00231569, NCT00280995, NCT00281008, NCT00362180, NCT00707746, NCT01090661, NCT00706849, NCT01299298, NCT01061814, NCT00794664, NCT01133366, NCT00694109, NCT00770146, NCT00607373, NCT01475825, NCT01414881, NCT00477594, NCT01598948	FDA, 2013	Heart, coronary artery	Subcutaneous injection	2006, 2010, 2012, 2015, 2016	31, 51, 52, 53, 54, 55, 56, 57
	PCSK9	Targeted knockdown	PCSK9 exon 12	siRNA, Mixed 2′‐fluoro and 2′‐O‐methyl‐PS	Phase 3 (Inclisiran)	NCT02963311, NCT01437059, NCT02597127, NCT02314442, NCT03705234, NCT03397121, NCT03400800, NCT03399370, NCT03851705, NCT03060577, NCT03159416, NCT03814187	No	Heart, coronary artery	Subcutanous injection	2011, 2014, 2016, 2017, 2018, 2019	58, 59, 60, 61, 62, 63, 64
Gaucher disease (231000)	GCS	Substrate reduction therapy (targeted knockdown)	GCS exon 8	siRNA (backbone not specified)	Preclinical	N.a.	No	Affects various tissues	N.a.	2006	65
Hereditary myopathy with lactic acidosis (255125)	ISCU	Exclusion of a variant‐induced pseudoexon	ISCU intron 5	2′‐O‐methyl‐PS	Preclinical	N.a.	No	Skeletal muscle	N.a.	2012	66
		Exclusion of a variant‐induced pseudoexon	ISCU intron 4	2′‐O‐methoxyethyl and Cet	Preclinical	N.a.	No	Skeletal muscle	N.a.	2016	67
Methylmalonic aciduria and Propionic acidaemia (251000 and 606054, respectively)	MUT, PCCA, PCCB	Exclusion of a variant‐induced pseudoexon	MUT intron 11, PCCA intron 14, PCCB intron 6	PMO	Preclinical	N.a.	No	Affects various tissues	N.a.	2007	68
Mucopolysaccharidosis type I and III (607015 and 252900)	EXTL2, EXTL3	Substrate reduction therapy (targeted knockdown)	Several targets in EXTL2 and EXTL3	shRNA expressed from U6 lentiviral vector	Preclinical	N.a.	No	Affects various tissues	N.a.	2010	69
Mucopolysaccharidosis type II (309900)	IDS	Blockage of an exonic cryptic splice site generated by a synonymous variant	IDS exon 8	PMO	Preclinical	N.a.	No	Affects various tissues	N.a.	2015	70, 71
Mucopolysaccharidosis type III (252900)	XYLT1, XYLT2, GALTI, GALTII,	Substrate reduction therapy (targeted knockdown)	Several targets in XYLT1, XYLT2, GALTI, GALTII	siRNA (backbone not specified)	Preclinical	N.a.	No	Affects various tissues	N.a.	2010	72
Niemann‐Pick type C1 (257220)	NPC1	Exclusion of a variant‐induced pseudoexon	NPC1 intron 9	2′‐O‐methoxyethyl	Preclinical	N.a.	No	Brain	N.a.	2009	73
Ocular Albinism Type I (300500)	GPR143	Exclusion of a variant‐induced pseudoexon	GPR143 intron 7	PMO	Preclinical	N.a.	No	Eye	N.a.	2006	74
Phenylketonuria (261600)	Slc6a19	Targeted knockdown	Slc6a19 intron 6	PMO	Preclinical	N.a.	No	Brain	N.a.	2018	75
Pompe disease (232300)	Gys1 (mouse)	Substrate reduction therapy (targeted knockdown)	Gys1 exon 6 splice donor	PMO	Preclinical	N.a.	No	Skeletal Muscle	N.a.	2014	76
	GAA	Blockage of several different natural cryptic splice sites	GAA exon 8, intron 10 and 15	PMO	Preclinical	N.a.	No	Skeletal Muscle	N.a.	2016	77
		Exclusion of a natural Pseudoexon	GAA intron 1	PMO	Preclinical	N.a.	No	Skeletal Muscle	N.a.	2017	78, 79
		Simultaneous blockage of different exonic splice silencers	GAA exon 2, multiple AONs	PMO	Preclinical	N.a.	No	Skeletal Muscle	N.a.	2017, 2019	80, 81
Primary hyperoxaluria type 1 and type 2 (259900 and 260000)	LDHA	Targeted knockdown	LDHA exon 7	siRNA, mixed 2′‐fluoro and 2′‐O‐methyl‐PS	Phase 3 (Nedosiran)	NCT04042402, NCT03392896, NCT03847909	No	Kidney	Subcutanous injection	2017, 2019	82, 83, 84
	HAO1	Targeted knockdown	HAO1 exon 8	siRNA, mixed 2′‐fluoro and 2′‐O‐methyl‐PS	Phase 3 (Lumasiran)	NCT02706886, NCT03350451, NCT03681184, NCT04125472, NCT03905694, NCT04152200	No	Kidney	Subcutanous injection	2016, 2017, 2018, 2019, 2020	84, 85, 86, 87
Pyridoxine‐dependent epilepsy (266100)	ALDH7A1	Blockage of an exonic cryptic splice site generated by a synonymous variant	ALDH7A1 exon 1	PMO	Preclinical	N.a.	No	Brain	N.a.	2013	88

### 4.1 Ongoing clinical trials with AONs for metabolic diseases

AON therapies under development for familial hypercholesterolemia (OMIM#607748) and primary hyperoxaluria type 1 and type 2 (OMIM#259900 and 260000, respectively) are currently tested in clinical trials.

While Mipomersen is an AON for the treatment of familial hypercholesterolemia that has been approved by the FDA, the EMA has not approved the use of this drug due to cardiovascular complications and heightened enzymatic liver values (https://www.ema.europa.eu/documents/assessment-report/kynamro-epar-public-assessment-report_en.pdf). These findings warranted the investigation of alternative treatments. Currently, an AON aimed at knockdown *PCSK9*, which encodes a protein that targets the LDL receptor for lysosomal degradation, is in phase III clinical trials and shows the ability to reduce cholesterol levels and has an acceptable safety profile.^34^, ^58^, ^59^, ^61^, ^63^, ^64^

Primary hyperoxaluria is caused by defects in the enzyme alanine‐glyoxylate aminotransferase (type 1, OMIM#259900) or glyoxylate reductase (type 2, OMIM#260000) leading to overproduction of oxalate. This results in severe kidney problems including calcium oxalate kidney stones, progressive oxalate nephropathy, and kidney failure. Advanced cases of the disease show systemic symptoms that can lead to severe morbidity and may eventually cause death. Knockdown of HAO1 (type 1) or LDHA (type 2) transcripts, which function upstream in the oxalate synthesis pathway using siRNA AONs reduces urinal oxalate content in preclinical studies, and early phase clinical trials. Phase 3 studies are currently underway for treatment of patients with primary hyperoxaluria type 1.^82^, ^83^, ^84^, ^85^, ^86^, ^87^

### 4.2 Preclinical AON studies for metabolic diseases

#### 4.2.1 AON‐mediated skipping of intronic variant‐induced pseudoexons

Splice modulating AONs have also been investigated in preclinical studies for inherited metabolic diseases. Interestingly, this mostly concerned studies where intronic disease‐associated variants generated a cryptic splice site that induced inclusion of a pseudoexon into the mRNA transcript. This disease mechanism has been exploited to develop a new treatment for several disorders based on blockage of the induced cryptic splice site (Table 2). As an example, erythropoietic protoporphyria (OMIM#177000) will be discussed here.^49^ This disease is caused by reduced levels of ferrochelatase protein, an enzyme involved in heme synthesis. When the ferrochelatase levels are below 35% of normal values, photosensitive protoporphyrin IX accumulates in red erythrocytes, plasma, skin, bile and feces. The most prominent clinical feature is photosensitivity. In over 90% of patients, this disease results from the inheritance of a mutated *FECH* allele in trans with a hypomorphic allele harboring a single nucleotide polymorphism (SNP) in intron 3. This SNP activates a pseudoexon that is included in a subset of transcripts. As such only reduced amounts of functional ferrochelatase protein are produced from this allele. Interestingly, since the vast majority of affected patients carry this SNP, a reduction of cryptic splicing should apply to most patients (Figure 2D). An AON targeting the pseudoexon has been developed and tested in patient derived cultured cells. It has also been tested in patient erythrocyte precursor cells, where treated cells showed a reduction in protoporphyrin IX accumulation.^49^

Several other metabolic disorders for which AON treatments are under development appear to involve exclusion of variant‐induced pseudoexons to restore normal splicing. These include 6‐pyruvoyl‐tetrahydropterin synthase deficiency (OMIM#261600), congenital disorder of glycosylation type 1a (CDG‐1a) (OMIM#212065), Fabry disease (OMIM#301500), hereditary myopathy with lactic acidosis (OMIM#255125), methylmalonic aciduria and propionic acidaemia (OMIM#251000 and 606054, respectively), Niemann‐Pick type C1 (OMIM#257220), and ocular albinism Type I (OMIM#300500) (Table 2).^44^, ^48^, ^49^, ^50^, ^66^, ^67^, ^68^, ^73^, ^74^ This highlights the relatively high prevalence of variant‐induced pseudoexon inclusion as a disease mechanism of metabolic disorders caused by missplicing. These types of variants appear to be promising candidates for restoring canonical splicing using an AON.

#### 4.2.2 Substrate‐reduction therapy using AONs as a potential treatment for metabolic disorders

Metabolic diseases including lysosomal storage diseases are characterized by the accumulation of substrates due to deficiency of specific metabolic enzymes.^90^ AON treatments that aim at reducing substrates using knockdown of genes that are important for production of the substrate, termed substrate reduction therapy (SRT), have been investigated for several diseases (Figure 1). Mucopolysaccharidosis type I and III (OMIM#607015 and 252900, respectively) are characterized by the accumulation of the polysaccharide heparan sulphate. Several studies have investigated the use of AONs to knock down genes involved in the production of heparan sulphate.^69^, ^72^ For Gaucher disease (OMIM#231000) knockdown of UDP‐glucose ceramide glucosyltransferase (*UGCG*) using an siRNA has been tested in an effort to reduce glycosphingolipid levels.^65^ SRT has also been applied for Pompe disease (OMIM#232300), aimed at reduction of cytoplasmic glycogen by knock down of glycogen synthase to reduce glycogen levels in lysosomes.^76^ Lastly, SRT for Canavan disease (OMIM#271900), a vacuolar leukodystrophy caused by accumulation of N‐acetyl‐l‐aspartate (NAA), has been developed that utilizes a gapmer design AON to knockdown a protein involved in NAA synthesis.^47^

#### 4.2.3 Other splice switching mechanisms utilized for metabolic disorders

Additional splicing variants for which AONs could be a treatment strategy have been studied, such as a variant in the *IDS* gene that resulted in the generation of a new splice acceptor site within exon 8 (Figure 2E). Consequently, the first part of exon 8 was not included in the mRNA, thus abolishing the production of idunorate‐2‐sulphatase (IDS) protein and resulting in mucopolysaccharidosis II, a lysosomal storage disease (OMIM#309900). Here AON treatment of cells expressing a minigene system that recapitulated the variant resulted in two types of transcripts, one where normal splicing was restored, and one where exon 8 was skipped completely.^70^, ^71^ A splice switching AON that utilizes a similar mechanism has been designed for the treatment of pyridoxine‐dependent epilepsy (OMIM#266100).^88^

Splice‐switching AONs have also been applied as a therapeutic strategy for Pompe disease. Pompe disease is a lysosomal storage disorder caused by variants in the *GAA* gene, thereby disrupting the production of the enzyme acid α‐glucosidase (GAA), which results in glycogen accumulation throughout the body, leading to progressive myopathy. Recombinant enzyme replacement therapy is an approved therapy for Pompe disease, but this treatments has several drawbacks, including a varying response to treatment and formation of neutralizing antibodies. Multiple AONs have been generated to correct aberrant splicing caused by several different rare variants present in the Pompe population.^77^ These variants resulted in utilization of natural or variant‐induced cryptic splice sites. The AONs in this study were designed to sterically block these cryptic splice sites, and showed an increase of canonical splicing in all cases. Although these AONs look promising, they can only be utilized in a small part of the Pompe disease population. Most effort has focused on restoring canonical splicing in patients with Pompe disease that carry the c.‐32‐13T>G splicing variant.^78^, ^79^, ^80^, ^81^ Approximately 80% of Caucasian childhood and adult onset patients carry this variant in intron 1, which decreases the recognition of the splice acceptor site of exon 2 by the splicing machinery (Figure 2F,G). This results in inclusion of an intronic natural pseudoexon. Since the splice donor site of the natural pseudoexon is relatively close to the canonical exon 2 acceptor, exon 2 is excluded in the majority (~85%) of transcripts. Since exon 2 contains the AUG start codon, these transcripts cannot produce functional protein. However, these patients can produce low levels of GAA from the low levels of canonically spliced transcripts, thus explaining the late onset of the disease. In fibroblasts and iPSC‐derived myotubes generated from these patients, it has been possible to almost fully restore exon 2 inclusion using AONs that target the splice sites of the physiological pseudoexon (Figure 2F). This resulted in restoration of GAA enzyme activity. In another strategy, AONs promoted exon 2 inclusion by targeting exon 2 itself, which most likely involve the suppression of exonic splice silencer elements^80^, ^81^ (Figure 2G). This approach may offer an alternative to enzyme replacement therapy for patients with the c.‐32‐13T>G splicing variant in the future.

## 5 CONCLUDING REMARKS

AON therapies are coming of age, with multiple approved drugs and tens of late phase clinical trials ongoing. AON therapies also offer promise for inherited metabolism diseases. When evaluating whether AON therapy could be an option for any disease, the target tissue is probably the most important consideration. Efficient delivery is possible for liver, the nervous system and also for the eye. For other tissues, this is more challenging. Furthermore, due to turnover and clearance of AONs, target transcripts and proteins, AONs have only transient effects and treatment has to be repeated. The frequency depends on the target tissue, but also the dynamics of the target transcript and proteins. With other therapeutic areas are also maturing, such as gene therapy, one now has a choice between therapeutic modalities. Which one is more optimal will have to be determined on a case by case basis and depends on several factors such as target tissue, severity of the disease and speed of disease progression. For AAV gene therapy preexisting immunity to the viral vector used would preclude patients from being treated. The older patients are the higher the chance that they have undergone a natural AAV infection and thus have already developed anti‐AAV antibodies. For AONs preexisting immunity is not an issue, although immune resonses to RNA‐based treatments should be considered. Gene therapy will generally restore a missing protein, while with AONs it is possible to restore a protein, but also to achieve knockdown. While gene therapy is generally perceived as a one‐time treatment, in some forms like AAV, delivered transgenes remain cytoplasmic, so they will be lost with turnover of tissue. Retreatment with AAV is not an option as yet, so in those cases, AON treatment could be an option once the therapeutic effects of gene therapy have been lost.

Currently approved AONs mainly target rare diseases and are expensive. These high costs are not something specific for AONs, but relate to the fact that development costs for rare disease drugs are often comparable to those for common diseases. Since the number of treated patients is lower, development costs per patients are higher. Due to the high costs, reimbursement and therefore access can be challenging. In addition, in the European Union, where EMA approved drugs have to be marketed in each member state individually, the time between approval and access can be long depending on the marketing requirements which vary for different countries. The international rare disease research consortium (IRDiRC) aims to address these and other challenges facing the rare disease therapy field.^91^

Many AONs require treatment in a hospital (eg, intrathecal and intravenous infusions), which leads to additional costs and burden to the health care system. Nevertheless, there are also AONs in development for common diseases, for example, in a phase 2 study, subcutaneous treatment with 300 mg inclisiran, a GalNac‐conjugated siRNA targeting PSK9 can reduce cholesterol of over 50% for 6 months with only minor side effects.^34^ If the efficacy and safety can be confirmed in the currently ongoing phase III study involving 15 000 individuals, inclisiran treatment may offer an alternative to statins. With 10 approved AONs, 3 of which in the last 6 months, and more AONs being tested in phases II and III clinical trials with favorable results so far, it seems that AON therapy is now coming of age as a therapeutic modality.

## CONFLICT OF INTEREST

A. A. R. discloses being employed by LUMC which has patents on exon skipping technology, some of which has been licensed to BioMarin and subsequently sublicensed to Sarepta. As co‐inventor of some of these patents A. A. R. is entitled to a share of royalties. A. A. R. further discloses being ad hoc consultant for PTC Therapeutics, Sarepta Therapeutics, CRISPR Therapeutics, Summit PLC, Alpha Anomeric, BioMarin Pharmaceuticals Inc., Eisai, Astra Zeneca, Santhera, Audentes, Global Guidepoint and GLG consultancy, Grunenthal, Wave and BioClinica, having been a member of the Duchenne Network Steering Committee (BioMarin) and being a member of the scientific advisory boards of ProQR, Sarepta Therapeutics, Silence Therapeutics and Philae Pharmaceuticals. Remuneration for these activities is paid to LUMC. LUMC also received speaker honoraria from PTC Therapeutics and BioMarin Pharmaceuticals and funding for contract research from Italpharmaco, Zakini and Alpha Anomeric. E. K., A. B. and W. W. P. P. declare no conflict of interest.

## AUTHOR CONTRIBUTIONS

Annemieke Aartsma‐Rus and Elsa C. Kuijper generated an outline for the contents, Annemieke Aartsma‐Rus wrote the manuscript, edited the summary and generated the tables, Elsa C. Kuijper generated the summary, edited the manuscript and prepared the Figures. Atze J. Bergsma and W.W.M. Pim Pijnappel provided the sections specifically on inborn errors of metabolism and edited the manuscript.

## References

[1] Stix G . Shutting down a gene. Antisense drug wins approval. Sci Am. 1998;279(46):50. 9841380 10.1038/scientificamerican1198-46b

[2] Hair P , Cameron F , McKeage K . Mipomersen sodium: first global approval. Drugs. 2013;73:487‐493. 23564617 10.1007/s40265-013-0042-2

[3] Aartsma‐Rus A , Krieg AM . FDA approves Eteplirsen for Duchenne muscular dystrophy: the next chapter in the Eteplirsen saga. Nucl Acid Ther. 2017;27:1‐3. 10.1089/nat.2016.0657 PMC5312460 27929755

[4] Aartsma‐Rus A . FDA approval of Nusinersen for spinal muscular atrophy makes 2016 the year of splice modulating oligonucleotides. Nucl Acid Ther. 2017;27:67‐69. 10.1089/nat.2017.0665 28346110

[5] Benson MD , Waddington‐Cruz M , Berk JL , et al. Inotersen treatment for patients with hereditary transthyretin amyloidosis. N Engl J Med. 2018;379:22‐31. 29972757 10.1056/NEJMoa1716793 PMC12611561

[6] Adams D , Gonzalez‐Duarte A , O'Riordan WD , et al. Patisiran, an RNAi therapeutic, for hereditary transthyretin amyloidosis. N Engl J Med. 2018;379:11‐21. 29972753 10.1056/NEJMoa1716153

[7] Hoy SM . Patisiran: first global approval. Drugs. 2018;78:1625‐1631. 30251172 10.1007/s40265-018-0983-6

[8] Yang X , Lee SR , Choi YS , et al. Reduction in lipoprotein‐associated apoC‐III levels following volanesorsen therapy: phase 2 randomized trial results. J Lipid Res. 2016;57:706‐713. 26848137 10.1194/jlr.M066399 PMC4808774

[9] Sardh E , Harper P , Balwani M , et al. Phase 1 trial of an RNA interference therapy for acute intermittent porphyria. N Engl J Med. 2019;380:549‐558. 30726693 10.1056/NEJMoa1807838

[10] Roshmi RR , Yokota T . Viltolarsen for the treatment of Duchenne muscular dystrophy. Drugs Today (Barc). 2019;55:627‐639. 31720560 10.1358/dot.2019.55.10.3045038

[11] Crooke ST . Molecular mechanisms of antisense oligonucleotides. Nucl Acid Ther. 2017;27:70‐77. 10.1089/nat.2016.0656 PMC5372764 28080221

[12] Crooke ST , Witztum JL , Bennett CF , Baker BF . RNA‐targeted therapeutics. Cell Metab. 2018;27:714‐739. 29617640 10.1016/j.cmet.2018.03.004

[13] Jarver P , O'Donovan L , Gait MJ . A chemical view of oligonucleotides for exon skipping and related drug applications. Nucl Acid Ther. 2014;24:37‐47. 10.1089/nat.2013.0454 PMC3923385 24171481

[14] Eckstein F . Phosphorothioates, essential components of therapeutic oligonucleotides. Nucl Acid Ther. 2014;24:374‐387. 10.1089/nat.2014.0506 25353652

[15] Godfrey C , Desviat LR , Smedsrod B , et al. Delivery is key: lessons learnt from developing splice‐switching antisense therapies. EMBO Mol Med. 2017;9:545‐557. 28289078 10.15252/emmm.201607199 PMC5412803

[16] Goyenvalle A , Griffith G , Babbs A , et al. Functional correction in mouse models of muscular dystrophy using exon‐skipping tricyclo‐DNA oligomers. Nat Med. 2015;21:270‐275. 25642938 10.1038/nm.3765

[17] Finkel RS , Chiriboga CA , Vajsar J , et al. Treatment of infantile‐onset spinal muscular atrophy with nusinersen: a phase 2, open‐label, dose‐escalation study. Lancet (London, England). 2016;388:3017‐3026. 10.1016/S0140-6736(16)31408-8 27939059

[18] Rigo F , Chun SJ , Norris DA , et al. Pharmacology of a central nervous system delivered 2'‐O‐methoxyethyl‐modified survival of motor neuron splicing oligonucleotide in mice and nonhuman primates. J Pharmacol Exp Ther. 2014;350:46‐55. 24784568 10.1124/jpet.113.212407 PMC4056267

[19] Kulkarni JA , Cullis PR , van der Meel R . Lipid nanoparticles enabling gene therapies: from concepts to clinical utility. Nucl Acid Ther. 2018;28:146‐157. 10.1089/nat.2018.0721 29683383

[20] Nair JK , Willoughby JL , Chan A , et al. Multivalent N‐acetylgalactosamine‐conjugated siRNA localizes in hepatocytes and elicits robust RNAi‐mediated gene silencing. J Am Chem Soc. 2014;136:16958‐16961. 25434769 10.1021/ja505986a

[21] Hartmann G . Nucleic acid immunity. Adv Immunol. 2017;133:121‐169. 28215278 10.1016/bs.ai.2016.11.001 PMC7112058

[22] Krieg AM . Mechanisms and applications of immune stimulatory CpG oligodeoxynucleotides. Biochim Biophys Acta. 1999;1489:107‐116. 10807001 10.1016/s0167-4781(99)00147-5

[23] Krieg AM , Matson S , Fisher E . Oligodeoxynucleotide modifications determine the magnitude of B cell stimulation by CpG motifs. Antisense Nucleic Acid Drug Dev. 1996;6:133‐139. 8843328 10.1089/oli.1.1996.6.133

[24] Frazier KS . Antisense oligonucleotide therapies: the promise and the challenges from a toxicologic pathologist's perspective. Toxicol Pathol. 2015;43:78‐89. 25385330 10.1177/0192623314551840

[25] Levin AA . A review of the issues in the pharmacokinetics and toxicology of phosphorothioate antisense oligonucleotides. Biochim Biophys Acta. 1999;1489:69‐84. 10806998 10.1016/s0167-4781(99)00140-2

[26] Henry SP , Giclas PC , Leeds J , et al. Activation of the alternative pathway of complement by a phosphorothioate oligonucleotide: potential mechanism of action. J Pharmacol Exp Ther. 1997;281:810‐816. 9152389

[27] Sheehan JP , Phan TM . Phosphorothioate oligonucleotides inhibit the intrinsic tenase complex by an allosteric mechanism. Biochemistry. 2001;40:4980‐4989. 11305914 10.1021/bi002396x

[28] Crooke ST , Baker BF , Witztum JL , et al. The effects of 2'‐O‐Methoxyethyl containing antisense oligonucleotides on platelets in human clinical trials. Nucl Acid Ther. 2017;27:121‐129. 10.1089/nat.2016.0650 PMC5467133 28145801

[29] Levin AA . Treating disease at the RNA level with oligonucleotides. N Engl J Med. 2019;380:57‐70. 30601736 10.1056/NEJMra1705346

[30] Hausen P , Stein H . Ribonuclease H. An enzyme degrading the RNA moiety of DNA‐RNA hybrids. Eur J Biochem. 1970;14:278‐283. 5506170 10.1111/j.1432-1033.1970.tb00287.x

[31] McGowan MP , Tardif JC , Ceska R , et al. Randomized, placebo‐controlled trial of mipomersen in patients with severe hypercholesterolemia receiving maximally tolerated lipid‐lowering therapy. PLoS One. 2012;7:e49006. 23152839 10.1371/journal.pone.0049006 PMC3496741

[32] Won JI , Zhang J , Tecson KM , McCullough PA . Balancing low‐density lipoprotein cholesterol reduction and hepatotoxicity with Lomitapide mesylate and mipomersen in patients with homozygous familial hypercholesterolemia. Rev Cardiovasc Med. 2017;18:21‐28. 28509890 10.3909/ricm0834

[33] Doench JG , Petersen CP , Sharp PA . siRNAs can function as miRNAs. Genes Dev. 2003;17:438‐442. 12600936 10.1101/gad.1064703 PMC195999

[34] Watts JK , Ockene IS . RNA interference for the masses? siRNA targeting PCSK9 promises prevention of cardiovascular disease. Nucl Acid Ther. 2020;30:1‐3. 10.1089/nat.2019.0835 31928497

[35] Spitali P , Aartsma‐Rus A . Splice modulating therapies for human disease. Cell. 2012;148:1085‐1088. 22424220 10.1016/j.cell.2012.02.014

[36] Singh NN , Howell MD , Androphy EJ , Singh RN . How the discovery of ISS‐N1 led to the first medical therapy for spinal muscular atrophy. Gene Ther. 2017;24:520‐526. 28485722 10.1038/gt.2017.34 PMC5623086

[37] Lefebvre S , Burglen L , Reboullet S , et al. Identification and characterization of a spinal muscular atrophy‐determining gene. Cell. 1995;80:155‐165. 7813012 10.1016/0092-8674(95)90460-3

[38] Finkel RS , Mercuri E , Darras BT , et al. Nusinersen versus sham control in infantile‐onset spinal muscular atrophy. N Engl J Med. 2017;377:1723‐1732. 29091570 10.1056/NEJMoa1702752

[39] Mercuri E , Darras BT , Chiriboga CA , et al. Nusinersen versus sham control in later‐onset spinal muscular atrophy. N Engl J Med. 2018;378:625‐635. 29443664 10.1056/NEJMoa1710504

[40] De Vivo DC , Bertini E , Swoboda KJ , et al. Nusinersen initiated in infants during the presymptomatic stage of spinal muscular atrophy: interim efficacy and safety results from the phase 2 NURTURE study. Neuromusclar Disord. 2019;29:842‐856. 10.1016/j.nmd.2019.09.007 PMC7127286 31704158

[41] Schorling DC , Pechmann A , Kirschner J . Advances in treatment of spinal muscular atrophy ‐ new phenotypes, new challenges, new implications for care. J Neuromuscular Dis. 2020;7:1‐13. 10.3233/JND-190424 PMC7029319 31707373

[42] Niks EH , Aartsma‐Rus A . Exon skipping: a first in class strategy for Duchenne muscular dystrophy. Expert Opin Biol Ther. 2017;17:225‐236. 27936976 10.1080/14712598.2017.1271872

[43] Kim J , Hu C , Moufawad El Achkar C , et al. Patient‐customized oligonucleotide therapy for a rare genetic disease. N Engl J Med. 2019;381:1644‐1652. 31597037 10.1056/NEJMoa1813279 PMC6961983

[44] Brasil S , Viecelli HM , Meili D , et al. Pseudoexon exclusion by antisense therapy in 6‐pyruvoyl‐tetrahydropterin synthase deficiency. Hum Mutat. 2011;32:1019‐1027. 21542064 10.1002/humu.21529

[45] Agarwal S , Simon AR , Goel V , et al. Pharmacokinetics and pharmacodynamics of the small interfering ribonucleic acid, Givosiran, in patients with acute hepatic porphyria. Clin Pharmacol Ther. 2020 10.1002/cpt.1802. 31994716

[46] Scott LJ . Givosiran: first approval. Drugs. 2020;80:335‐339. 32034693 10.1007/s40265-020-01269-0

[47] Hull V , Wang Y , Burns T , et al. Antisense oligonucleotide reverses leukodystrophy in Canavan disease mice. Ann Neurol. 2020;87:480‐485. 31925837 10.1002/ana.25674 PMC8523037

[48] Vega AI , Perez‐Cerda C , Desviat LR , Matthijs G , Ugarte M , Perez B . Functional analysis of three splicing mutations identified in the PMM2 gene: toward a new therapy for congenital disorder of glycosylation type Ia. Hum Mutat. 2009;30:795‐803. 19235233 10.1002/humu.20960

[49] Oustric V , Manceau H , Ducamp S , et al. Antisense oligonucleotide‐based therapy in human erythropoietic protoporphyria. Am J Hum Genet. 2014;94:611‐617. 24680888 10.1016/j.ajhg.2014.02.010 PMC3980518

[50] Palhais B , Dembic M , Sabaratnam R , et al. The prevalent deep intronic c. 639+919 G>A GLA mutation causes pseudoexon activation and Fabry disease by abolishing the binding of hnRNPA1 and hnRNP A2/B1 to a splicing silencer. Mol Genet Metab. 2016;119:258‐269. 27595546 10.1016/j.ymgme.2016.08.007

[51] Kastelein JJ , Wedel MK , Baker BF , et al. Potent reduction of apolipoprotein B and low‐density lipoprotein cholesterol by short‐term administration of an antisense inhibitor of apolipoprotein B. Circulation. 2006;114:1729‐1735. 17030687 10.1161/CIRCULATIONAHA.105.606442

[52] Raal FJ , Santos RD , Blom DJ , et al. Mipomersen, an apolipoprotein B synthesis inhibitor, for lowering of LDL cholesterol concentrations in patients with homozygous familial hypercholesterolaemia: a randomised, double‐blind, placebo‐controlled trial. Lancet (London, England). 2010;375:998‐1006. 10.1016/S0140-6736(10)60284-X 20227758

[53] Stein EA , Dufour R , Gagne C , et al. Apolipoprotein B synthesis inhibition with mipomersen in heterozygous familial hypercholesterolemia: results of a randomized, double‐blind, placebo‐controlled trial to assess efficacy and safety as add‐on therapy in patients with coronary artery disease. Circulation. 2012;126:2283‐2292. 23060426 10.1161/CIRCULATIONAHA.112.104125

[54] Visser ME , Wagener G , Baker BF , et al. Mipomersen, an apolipoprotein B synthesis inhibitor, lowers low‐density lipoprotein cholesterol in high‐risk statin‐intolerant patients: a randomized, double‐blind, placebo‐controlled trial. Eur Heart J. 2012;33:1142‐1149. 22507979 10.1093/eurheartj/ehs023 PMC3751967

[55] Flaim JD , Grundy JS , Baker BF , McGowan MP , Kastelein JJ . Changes in mipomersen dosing regimen provide similar exposure with improved tolerability in randomized placebo‐controlled study of healthy volunteers. J Am Heart Assoc. 2014;3:e000560. 24627419 10.1161/JAHA.113.000560 PMC4187476

[56] Santos RD , Duell PB , East C , et al. Long‐term efficacy and safety of mipomersen in patients with familial hypercholesterolaemia: 2‐year interim results of an open‐label extension. Eur Heart J. 2015;36:566‐575. 24366918 10.1093/eurheartj/eht549 PMC4344956

[57] Duell PB , Santos RD , Kirwan BA , Witztum JL , Tsimikas S , Kastelein JJP . Long‐term mipomersen treatment is associated with a reduction in cardiovascular events in patients with familial hypercholesterolemia. J Clin Lipidol. 2016;10:1011‐1021. 27578134 10.1016/j.jacl.2016.04.013

[58] Fitzgerald K , Frank‐Kamenetsky M , Shulga‐Morskaya S , et al. Effect of an RNA interference drug on the synthesis of proprotein convertase subtilisin/kexin type 9 (PCSK9) and the concentration of serum LDL cholesterol in healthy volunteers: a randomised, single‐blind, placebo‐controlled, phase 1 trial. Lancet (London, England). 2014;383:60‐68. 10.1016/S0140-6736(13)61914-5 PMC4387547 24094767

[59] Bandyopadhyay D , Qureshi A , Ghosh S , et al. Safety and efficacy of extremely low LDL‐cholesterol levels and its prospects in hyperlipidemia management. J Lipids. 2018;2018:8598054. 29850255 10.1155/2018/8598054 PMC5937425

[60] Chandra Ghosh G , Bandyopadhyay D , Ghosh RK , Mondal S , Herzog E . Effectiveness and safety of Inclisiran, a novel long‐acting RNA therapeutic inhibitor of Proprotein convertase subtilisin/kexin 9. Am J Cardiol. 2018;122:1272‐1277. 30075894 10.1016/j.amjcard.2018.06.023

[61] Leiter LA , Teoh H , Kallend D , et al. Inclisiran lowers LDL‐C and PCSK9 irrespective of diabetes status: the ORION‐1 randomized clinical trial. Diabetes Care. 2019;42:173‐176. 30487231 10.2337/dc18-1491

[62] Ray KK , Stoekenbroek RM , Kallend D , et al. Effect of 1 or 2 doses of Inclisiran on low‐density lipoprotein cholesterol levels: one‐year follow‐up of the ORION‐1 randomized clinical trial. JAMA Cardiol. 2019 10.1001/jamacardio.2019.3502. PMC6763983 31553410

[63] Raal FJ , Kallend D , Ray KK , et al. Inclisiran for the treatment of heterozygous familial hypercholesterolemia. N Engl J Med. 2020;382:1520‐1530. 32197277 10.1056/NEJMoa1913805

[64] Ray KK , Wright RS , Kallend D , et al. Two phase 3 trials of Inclisiran in patients with elevated LDL cholesterol. N Engl J Med. 2020;382:1507‐1519. 32187462 10.1056/NEJMoa1912387

[65] Diaz‐Font A , Chabas A , Grinberg D , Vilageliu L . RNAi‐mediated inhibition of the glucosylceramide synthase (GCS) gene: a preliminary study towards a therapeutic strategy for Gaucher disease and other glycosphingolipid storage diseases. Blood Cells Mol Dis. 2006;37:197‐203. 16959503 10.1016/j.bcmd.2006.07.002

[66] Sanaker PS , Toompuu M , McClorey G , Bindoff LA . Antisense oligonucleotide corrects splice abnormality in hereditary myopathy with lactic acidosis. Gene. 2012;494:231‐236. 22155317 10.1016/j.gene.2011.11.021

[67] Holmes‐Hampton GP , Crooks DR , Haller RG , et al. Use of antisense oligonucleotides to correct the splicing error in ISCU myopathy patient cell lines. Hum Mol Genet. 2016;25:5178‐5187. 28007899 10.1093/hmg/ddw338 PMC6078641

[68] Rincon A , Aguado C , Desviat LR , Sanchez‐Alcudia R , Ugarte M , Perez B . Propionic and methylmalonic acidemia: antisense therapeutics for intronic variations causing aberrantly spliced messenger RNA. Am J Hum Genet. 2007;81:1262‐1270. 17966092 10.1086/522376 PMC2276355

[69] Kaidonis X , Liaw WC , Roberts AD , Ly M , Anson D , Byers S . Gene silencing of EXTL2 and EXTL3 as a substrate deprivation therapy for heparan sulphate storing mucopolysaccharidoses. Eur J Hum Genet. 2010;18:194‐199. 19690583 10.1038/ejhg.2009.143 PMC2987189

[70] Matos L , Goncalves V , Pinto E , et al. Data in support of a functional analysis of splicing mutations in the IDS gene and the use of antisense oligonucleotides to exploit an alternative therapy for MPS II. Data Brief. 2015;5:810‐817. 26693516 10.1016/j.dib.2015.10.011 PMC4660375

[71] Matos L , Goncalves V , Pinto E , et al. Functional analysis of splicing mutations in the IDS gene and the use of antisense oligonucleotides to exploit an alternative therapy for MPS II. Biochim Biophys Acta. 2015;1852:2712‐2721. 26407519 10.1016/j.bbadis.2015.09.011

[72] Dziedzic D , Wegrzyn G , Jakobkiewicz‐Banecka J . Impairment of glycosaminoglycan synthesis in mucopolysaccharidosis type IIIA cells by using siRNA: a potential therapeutic approach for Sanfilippo disease. Eur J Hum Genet. 2010;18:200‐205. 19690584 10.1038/ejhg.2009.144 PMC2987185

[73] Rodriguez‐Pascau L , Coll MJ , Vilageliu L , Grinberg D . Antisense oligonucleotide treatment for a pseudoexon‐generating mutation in the NPC1 gene causing Niemann‐Pick type C disease. Hum Mutat. 2009;30:E993‐E1001. 19718781 10.1002/humu.21119

[74] Vetrini F , Tammaro R , Bondanza S , et al. Aberrant splicing in the ocular albinism type 1 gene (OA1/GPR143) is corrected in vitro by morpholino antisense oligonucleotides. Hum Mutat. 2006;27:420‐426. 16550551 10.1002/humu.20303

[75] Belanger AM , Przybylska M , Gefteas E , et al. Inhibiting neutral amino acid transport for the treatment of phenylketonuria. JCI Insight. 2018;3:121762 30046012 10.1172/jci.insight.121762 PMC6124451

[76] Clayton NP , Nelson CA , Weeden T , et al. Antisense oligonucleotide‐mediated suppression of muscle glycogen synthase 1 synthesis as an approach for substrate reduction therapy of Pompe disease. Mol Ther Nucl Acids. 2014;3:e206. 10.1038/mtna.2014.57 PMC4217081 25350581

[77] Bergsma AJ , In't Groen SL , Verheijen FW , van der Ploeg AT , Pijnappel W . From cryptic toward canonical pre‐mRNA splicing in Pompe disease: a pipeline for the development of antisense oligonucleotides. Mol Ther Nucl Acids. 2016;5:e361. 10.1038/mtna.2016.75 PMC5056997 27623443

[78] van der Wal E , Bergsma AJ , Pijnenburg JM , van der Ploeg AT , Pijnappel W . Antisense oligonucleotides promote exon inclusion and correct the common c.‐32‐13T>G GAA splicing variant in Pompe disease. Mol Ther Nucl Acids. 2017;7:90‐100. 10.1016/j.omtn.2017.03.001 PMC5415969 28624228

[79] van der Wal E , Bergsma AJ , van Gestel TJM , et al. GAA deficiency in Pompe disease is alleviated by exon inclusion in iPSC‐derived skeletal muscle cells. Mol Ther Nucl Acids. 2017b;7:101‐115. 10.1016/j.omtn.2017.03.002 PMC5415960 28624186

[80] Goina E , Peruzzo P , Bembi B , Dardis A , Buratti E . Glycogen reduction in myotubes of late‐onset Pompe disease patients using antisense technology. Mol Ther. 2017;25:2117‐2128. 28629821 10.1016/j.ymthe.2017.05.019 PMC5589062

[81] Goina E , Musco L , Dardis A , Buratti E . Assessment of the functional impact on the pre‐mRNA splicing process of 28 nucleotide variants associated with Pompe disease in GAA exon 2 and their recovery using antisense technology. Hum Mutat. 2019;40:2121‐2130. 31301153 10.1002/humu.23867

[82] Dutta C , Avitahl‐Curtis N , Pursell N , et al. Inhibition of glycolate oxidase with dicer‐substrate siRNA reduces calcium oxalate deposition in a mouse model of primary hyperoxaluria type 1. Mol Ther. 2016;24:770‐778. 26758691 10.1038/mt.2016.4 PMC4886950

[83] Lai C , Pursell N , Gierut J , et al. Specific inhibition of hepatic lactate dehydrogenase reduces oxalate production in mouse models of primary hyperoxaluria. Mol Ther. 2018;26:1983‐1995. 29914758 10.1016/j.ymthe.2018.05.016 PMC6094358

[84] Milliner DS , McGregor TL , Thompson A , et al. Endpoints for clinical trials in primary hyperoxaluria. Clin J Am Soc Nephrol. 2020;CJN.13821119. 10.2215/CJN.13821119 PMC7341772 32165440

[85] Li X , Knight J , Fargue S , et al. Metabolism of (13)C5‐hydroxyproline in mouse models of primary hyperoxaluria and its inhibition by RNAi therapeutics targeting liver glycolate oxidase and hydroxyproline dehydrogenase. Biochim Biophys Acta. 2016;1862:233‐239. 26655602 10.1016/j.bbadis.2015.12.001 PMC4706777

[86] Liebow A , Li X , Racie T , et al. An investigational RNAi therapeutic targeting glycolate oxidase reduces oxalate production in models of primary hyperoxaluria. J Am Soc Nephrol. 2017;28:494‐503. 27432743 10.1681/ASN.2016030338 PMC5280024

[87] Wood KD , Holmes RP , Erbe D , Liebow A , Fargue S , Knight J . Reduction in urinary oxalate excretion in mouse models of primary Hyperoxaluria by RNA interference inhibition of liver lactate dehydrogenase activity. Biochim Biophys Acta Mol Basis Dis. 2019;1865:2203‐2209. 31055082 10.1016/j.bbadis.2019.04.017 PMC6613992

[88] Perez B , Gutierrez‐Solana LG , Verdu A , et al. Clinical, biochemical, and molecular studies in pyridoxine‐dependent epilepsy. Antisense therapy as possible new therapeutic option. Epilepsia. 2013;54:239‐248. 23350806 10.1111/epi.12083

[89] Perez B , Vilageliu L , Grinberg D , Desviat LR . Antisense mediated splicing modulation for inherited metabolic diseases: challenges for delivery. Nucl Acid Ther. 2014;24:48‐56. 10.1089/nat.2013.0453 PMC3922136 24506780

[90] Coutinho MF , Santos JI , Alves S . Less is more: substrate reduction therapy for lysosomal storage disorders. Int J Mol Sci. 2016;17:E1065 27384562 10.3390/ijms17071065 PMC4964441

[91] Austin CP , Cutillo CM , Lau LPL , et al. Future of rare diseases research 2017‐2027: an IRDiRC perspective. Clin Transl Sci. 2018;11:21‐27. 28796445 10.1111/cts.12500 PMC5759721

